# Text Data Augmentation for Deep Learning

**DOI:** 10.1186/s40537-021-00492-0

**Published:** 2021-07-19

**Authors:** Connor Shorten, Taghi M. Khoshgoftaar, Borko Furht

**Affiliations:** Florida Atlantic University, 777 Glades Road, Boca Raton, FL 33431 USA

**Keywords:** Data Augmentation, Natural Language Processing, Overfitting, Big Data, NLP, Text Data

## Abstract

Natural Language Processing (NLP) is one of the most captivating applications of Deep Learning. In this survey, we consider how the Data Augmentation training strategy can aid in its development. We begin with the major motifs of Data Augmentation summarized into strengthening local decision boundaries, brute force training, causality and counterfactual examples, and the distinction between meaning and form. We follow these motifs with a concrete list of augmentation frameworks that have been developed for text data. Deep Learning generally struggles with the measurement of generalization and characterization of overfitting. We highlight studies that cover how augmentations can construct test sets for generalization. NLP is at an early stage in applying Data Augmentation compared to Computer Vision. We highlight the key differences and promising ideas that have yet to be tested in NLP. For the sake of practical implementation, we describe tools that facilitate Data Augmentation such as the use of consistency regularization, controllers, and offline and online augmentation pipelines, to preview a few. Finally, we discuss interesting topics around Data Augmentation in NLP such as task-specific augmentations, the use of prior knowledge in self-supervised learning versus Data Augmentation, intersections with transfer and multi-task learning, and ideas for AI-GAs (AI-Generating Algorithms). We hope this paper inspires further research interest in Text Data Augmentation.

## Introduction

Nearly all the successes of Deep Learning stem from supervised learning. Supervised learning describes the use of loss functions that align predictions with manually annotated ground truth. Deep Learning can achieve remarkable performance through the combination of this learning strategy and large labeled datasets. The problem is that collecting these annotated datasets is very difficult at the scale required. For example, one of the key Deep Learning applications for COVID-19 rapid response was question answering [1]. Tang et al. [2] constructed COVID-QA, a supervised learning dataset in which articles are annotated with an answer span to a given question. The authors of the paper describe working for 23 hours to produce 124 question-answer pairs. Fitting 124 question-answer annotations without overfitting is extremely challenging in the current state of Deep Learning. In addition to question answering, Natural Language Processing (NLP) researchers are also exploring the application of abstractive summarization in which a model outputs a novel summary from a collection of input documents. Cachola et al. [3] were able to collect a dataset of 5.4K Too Long; Didn’t Read (TLDR) summaries of 3.2K machine learning papers. This required employing 28 undergraduate students to refine data bootstrapped from the OpenReview platform. These anecdotes are provided to highlight the difficulty of curating annotated big data for knowledge-intensive NLP tasks with millions of examples.

The Deep Learning research community is currently exploring many solutions to the problem of learning without labeled big data. In addition to Data Augmentation, self-supervised learning and transfer learning have performed very well. Few and zero-shot learning are categories of research gaining interest as well. In this survey, we explore getting more performance out of the supervised data available with Data Augmentation. Our survey additionally explores how Data Augmentation is driving key advances in learning strategies outside of supervised learning. This includes self-supervised learning from unlabeled datasets, and transfer learning from other domains, whether that data is labeled or unlabeled.

Data Augmentation describes a set of algorithms that construct synthetic data from an available dataset. This synthetic data typically contains small changes in the data that the model’s predictions should be invariant to. Synthetic data can also represent combinations between distant examples that would be very difficult to infer otherwise. Data Augmentation is one of the most useful interfaces to influence the training of Deep Neural Networks. This is largely due to the interpretable nature of the transformations and the window to observe how the model is failing.

Preventing overfitting is the most common use case of Data Augmentation. Without augmentation, or regularization more generally, Deep Neural Networks are prone to learning spurious correlations and memorizing high-frequency patterns that are difficult for humans to detect. In NLP, this could describe high frequency numeric patterns in token embeddings, or memorizations of particular forms of language that do not generalize. Data Augmentation can aid in these types of overfitting by shuffling the particular forms of language. To overcome the noisy data, the model must resort to learning abstractions of information which are more likely to generalize.

Data Augmentation is a regularization strategy. Other regularization techniques have been developed such as dropout [4] or weight penalties [5]. These techniques apply functional regularization by either adding noise to intermediate activations of the network or adding constraints to the functional form. These techniques have found successes, but they lack the power to express the esoteric concept of semantic invariance. Data Augmentation enables an intuitive interface for demonstrating label-preserving transformations.

Our survey presents several strategies for applying Data Augmentation to text data. We cluster these augmentations into symbolic or neural methods. Symbolic methods use rules or discrete data structures to form synthetic examples. This includes Rule-Based Augmentations, Graph-Structured Augmentations, Feature-Space Augmentation, and MixUp. Neural augmentations use a deep neural network trained on a different task to augment data. Neural augmentations surveyed include Back-Translation, Generative Data Augmentation, and Style Augmentation. In addition to symbolic vs. neural-based augmentations, we highlight other distinctions between augmentations such as task-specific versus task-agnostic augmentations and form versus meaning augmentations. We describe these distinctions further throughout our survey.

Generalization is the core challenge of Deep Learning. How far can we extrapolate from the instances available? The same interface used to control the training data is also useful for simulating potential test sets and distribution shifts. We can simulate distribution shift by applying augmentations to a dataset, such as adding random tokens to an email spam detector or increasing the prevalence of tokens that lie on the long-tail of the frequency distributions. These simulated shifts can also describe higher-level linguistic phenomenon. This involves deeper fact chaining than what was seen in the training set, or the ability to change predictions given counterfactual evidence. As our tools for Generative Data Augmentation continue to improve, we will be able to simulate more semantic distribution shifts. This looks like a very promising direction to advance generalization testing.

Our survey on Text Data Augmentation for Deep Learning builds on our work surveying Image Data Augmentation for Deep Learning [6]. In Computer Vision, this describes applying transformations such as rotating images, horizontally flipping them, or increasing the brightness to form augmented examples. We found that it is currently much easier to apply label-preserving transformations in Computer Vision than NLP. It is additionally easier to stack these augmentations in Computer Vision, enabling even more diversity in the augmented set, which has been shown to be a key contributor to success. Data Augmentation research has been more thoroughly explored in Computer Vision than NLP. We present some ideas that have found interesting results with images, but remain to be tested in the text data domain. Finally, we discuss the intersection of visual supervision for language understanding and how vision-language models may help overcome the grounding problem. We discuss the grounding problem in greater detail under our Motifs Of Data Augmentation section.

Our next section presents practical implementation decisions for text data augmentation. We begin by describing the use of a consistency regularization loss to further influence the impact of augmented data. Differently from consistency regularization, contrastive learning additionally uses negative examples to structure the loss function. The next key question is how to control the strength and sampling of each augmentation. Augmentation controllers apply a meta-level abstraction to the hyperparameters of augmentation selection and the magnitude of the transformation. This is commonly explored with an adversarial controller that aims to produce mistakes in the model. We also describe controllers that search for performance improvements such as AutoAugment [7], Population-Based Augmentation [8], and RandAugment [9]. Although similar in concept, we discuss the key distinction between augmentation controllers and curriculum learning. Another important consideration for implementing Data Augmentation is the CPU to GPU transfer in the preprocessing pipeline, as well as the conceptual understanding of offline versus online augmentation. Finally, we describe the application of augmentation to alleviate issues caused by class imbalance.

Our Discussion section presents opportunities to explore text data augmentation. We begin with task-specific augmentations describing how key NLP tasks such as question answering differ from natural language inference, particularly with respect to input length or the categorization as a knowledge-intensive task. We quickly previewed that self-supervised and transfer learning are also emerging solutions to learning with limited labeled data. We discuss the use of Data Augmentation in self-supervised learning and then recent works with transfer and multi-task learning. Finally, we discuss AI-GAs, short for AI-generating Algorithms [10]. This is a very interesting idea encompassing papers such as POET [11], Generative Teaching Networks [12], and the Synthetic Petri Dish [13] which describe algorithms that learn the environment to learn from. We present how this differs from augmentation controllers or curriculum learning, the idea of skill acquisition from artificial data, and opportunities to test these ideas in NLP.

Data Augmentation for NLP prevents overfitting, provides the easiest way to inject prior knowledge into a Deep Learning system, and offers a view into the generalization ability of these models. Our survey is organized as follows:

- We begin with the key Motifs Of Data Augmentation that augmentations strive to achieve.
- We provide a list of Text Data Augmentations. This list can be summarized into symbolic augmentations, using rules and graph-structured decomposition to form new examples, and neural augmentations, that use auxiliary neural networks to sample new data.
- Following our list of available augmentations, we dive deeper into Testing Generalization with Data Augmentation.
- We continue with a comparison of Image versus Text Augmentation.
- Returning to Text Data Augmentation, we describe Practical Considerations for Implementation.
- Finally, we present interesting ideas and research questions in our Discussion section.
- Our Conclusion briefly summarizes the motivation and findings of our survey.

## Background

Data Augmentation has been a heavily studied area of Machine Learning. The advancement of the prior knowledge encoded in augmentations is one of the key distinctions between previous works and now. As we will discuss in depth later in the survey, the success of Data Augmentation in Computer Vision has been fueled by the ease of designing label-preserving transformations. For example, a cat image is still a cat after rotating it, translating it on the x or y axis, increasing the intensity of the red channel, and so on. It is easy to brainstorm these semantically-preserving augmentations for images, whereas it is much harder to do this in the text domain.

We believe our survey on text data augmentation is well-timed with respect to questions such as why now? What has changed recently? Recent advances in generative modeling such as StyleGAN for images, GPT-3 for text [14], and DALL-E unifying both text and images [15], have been astounding. We summarize many exciting works on the use of prompting for adapting language models for downstream tasks. As discussed in further detail later on, we believe these advances in generative modeling could be game changing for the way we store datasets and build Deep Learning models. More particularly, it could become common to use labeled datasets solely for the sake of evaluation, rather than representation learning.

Our survey has some similarities to Feng et al. [16] which has been published roughly around the same time as ours. Both surveys seek a clear definition of Data Augmentation and aim to highlight key motifs. Additionally, both surveys narrate the development of NLP augmentation around the successes of augmentation in Computer Vision and how these may transfer. Feng et al. [16] provide a deeper enumeration of task-specific augmentation than is covered in our survey. Our survey adds important concepts such as the debate between Meaning versus Form, Counterfactual Examples, and the use of prompts in Generative Data Augmentation.

Many of the successes of Deep Learning stem from access to large labeled datasets such as ImageNet [17]. However, constructing these datasets is very challenging and time-consuming. Therefore, researchers are looking for alternative ways to leverage data without manual annotation. This is a large motivation behind the success of self-supervised language modeling with papers such as GPT-3 [14] or BERT [18]. Data Augmentation follows this same motivation as overcoming the challenge of learning with limited labeled data and avoiding manually labeling data. For example, many of the surveyed studies highlight the success of their algorithms when sub-setting the labeled data.

Transfer Learning has been one of the most effective solutions to this challenge of learning from limited labeled datasets [19]. Transfer Learning references initialization of the model for learning with the weights learned from a previous task. This previous task usually has the benefit of big data, whether that data is labeled such as ImageNet or unlabeled, as is used in self-supervised language models. There are many research questions around the procedure of Transfer Learning. In our Discussion section we discuss opportunities with Data Augmentation such as freezing the base feature extractor and training separate heads on the original and augmented datasets.

Self-supervised learning describes a general set of algorithms that learn from unlabeled data with supervised learning. This is done by algorithmically labeling the data. Some of the most popular self-supervised learning tasks include generation, contrastive learning, and pretext tasks. Generation describes how language models are trained. A token is algorithmically selected to be masked out and the masked out token is used as the label for supervised learning. Contrastive learning aligns representations of data algorithmically determined to be similar (usually through the use of augmentations), and distances these representations from negatives (usually other samples in the mini-batch). Pretext tasks describe ideas such as applying an augmentation to data and tasking the model to predict the transformation. The augmentation interface powers many task constructions in self-supervised learning.

## Motifs of text data augmentation

This section will introduce a unifying view of objective the augmentations presented in the rest of the survey address. We introduce the key motifs of Text Data Augmentation as Strengthening Decision Boundaries, Brute Force Training, Causality and Counterfactual Examples, and the distinction between Meaning versus Form. These concepts dig into the understanding of Data Augmentation and their particular application to language processing.

### Strengthening decision boundaries

Data Augmentation is commonly applied to classification problems where class boundaries are learned from label assignments. Augmented examples are typically only slightly different from existing samples. Training on these examples results in added space between the original example and its respective class boundary. Well defined class boundaries result in more robust classifiers and uncertainty estimates. For example, these boundaries are often reported with lower dimensional visualizations derived from t-SNE [20] or UMAP [21].

A key motif of Data Augmentation is to perturb data so that the model is more familiar with the local space around these examples. Expanding the radius from each example in the dataset will overall help the model get a better sense of the decision boundary and result in smother interpolation paths. This is in reference to small changes to the original data points. In NLP this could be deleting or adding words, synonym swaps, or well controlled paraphrases. The model becomes more robust to the local space and decision boundary based on available labels simply by increased exposure.

### Brute force training

Deep Neural Networks are highly parametric models with very high variance that can easily model their training data. Fitting the training data is surprisingly robust to interpolation, or moving within the data points provided. What Deep Learning struggles with, as we will unpack in Generalization Testing with Data Augmentation, is extrapolating outside of data points provided during training. A potential solution to this is to brute force the data space with the training data.

The upper bound solution to many problems in Computer Science is to simply enumerate all candidate solutions. Brute force solutions rely on computing speed to overpower the complexity of a given problem. In Deep Learning, this entails training on an exhaustive set of natural language sequences such that all potential distributions the test set could be sampled from are covered in the training data. This way, even the most extreme edge cases will have been covered in the training set. The design of brute force training requires exhaustive coverage of the natural language manifold. A key question is whether this idea is reasonable or not? It may be better to identify key regions that are missing, although that it is challenging to probe for and define.

### Causality and counterfactual examples

Vital to achieving the goals of Deep Learning, is to learn causal representations [22], as opposed to solely representing correlations. The field of Causal Inference demonstrates how to use interventions to establish causality. Reinforcement Learning is the most similar branch of Deep Learning research in which an agent deliberately samples interventions to learn about its environment. In this survey, we consider how the results of interventions can be integrated into observational language data. This is also similar to the subset of Reinforcement Learning known as the offline setting [23].

Many of the Text Data Augmentations described throughout the survey utilize the terminology of Counterfactual Examples [24]. These Counterfactual Examples describe augmentations such as the introduction of negations or numeric alterations to flip the label of the example. The construction of counterfactuals in language generally relies on human expertise, rather than algorithmic construction. Although the model does not deliberately sample these interventions akin to a randomized control trial, the hope is that it can still establish causal links between semantic concepts and labels by observing the result of interventions.

Liu et al. [25] lay the groundwork for formal causal language in Data Augmentation. This entails the use of structured causal models and the procedure of abduction, action, and prediction to generate counterfactual examples. These experiments rely on phrasal alignment between sequences in neural machine translation to sample counterfactual replacements. Their counterfactual augmentation improves on a baseline English to French translation system from 26.0 to 28.92 according to the BLEU metric. It seems possible that this phrasal alignment could be extended to other sequence-to-sequence problems such as abstractive question answering, summarization, or dialogue systems. This explicit counterfactual structure is different from most reviewed works that rather use natural language prompts to automate counterfactual sampling. For example, DINO [26] generates natural language inference data by either seeding the generation with “mean the same thing” or “are on completely different topics”. We think it is an interesting research direction to see if rigorous causal modeling such as computing the conditional probabilities of the context removing the variable [27] will provide benefits over prompts and large language models.

### Meaning versus form

One of the most interesting ideas in language processing is the distinction between meaning and form. Bender and Koller [28] introduced the argument, providing several ideas and thought experiments. A particularly salient anecdote to illustrate this is known as the octopus example. In this example, two people are stranded on separate islands, communicating through an underwater cable. This underwater cable is intercepted by an intelligent octopus who learns to mimic the speaking patterns of each person. The octopus does this well enough that it can substitute for either person, as in the Turing test. However, when one of the stranded islanders encounters a bear and seeks advice, the octopus is unable to help. This is because the octopus has learned the form of their communication, but it has not learned the underlying meaning of the world in which their language describes.

We will present many augmentations in this paper that aid in learning form. Similar to the concept of strengthening decision boundaries, ideas like synonym swap or rotating syntactic trees will help the octopus further strengthen its understanding of how language is generally organized. With respect to achieving an understanding of meaning in these models and defining this esoteric concept, many have turned to ideas in grounding and embodiment. Grounding typically refers to pairing language with other modalities such as vision-language or audio-language models. However, grounding can also refer to abstract concepts and worlds constructed solely from language. Embodiment references learning agents that act in their environment. Although Bender and Koller propose that meaning cannot be learned from form alone, many other works highlight different areas of the language modeling task such as assertions [29] or multiple embedded tasks [30] that could lead to learning meaning. Another useful way of thinking about meaning versus form could be to look at recently developed benchmarks in language processing such as the distinction between GLUE [31] and SuperGLUE [32] tasks that predominantly test an understanding of form to knowledge-intensive tasks such as KILT [33] that better probe for meaning. In our survey, we generally use the terms “understanding” and “meaning” to describe passing black-box tests designed by humans. We believe that drilling into the definition of these terms is one of the most promising pursuits in language processing research.

## Text data augmentations

We described Data Augmentation as a strategy to prevent overfitting via regularization. This regularization is enabled through an intuitive interface. As we study a task or dataset, we learn more about what kind of priors or what kind of additional data we need to collect to improve the system. For example, we might discover characteristics about our question answering dataset such as that it fails with symmetric consistency on comparison questions. The following list of augmentations describes the mechanisms we currently have available to inject these priors into our datasets.

### Symbolic augmentation

We categorize these augmentations as “Symbolic Augmentations” in contrast to “Neural Augmentations”. As stated earlier, the key difference is the use of auxiliary neural networks, or other types of statistical models, to generate data compared to using symbolic rules to augment data. A key benefit of symbolic augmentation is the interpretability for the human designer. Symbolic augmentations also work better with short transformations, such as replacing words or phrases to form augmented examples. However, some information-heavy applications rely on longer inputs such as question answering or summarization. Symbolic rules are limited in applying global transformations such as augmenting entire sentences or paragraphs.

#### Rule-based augmentation

**Fig. 1:**
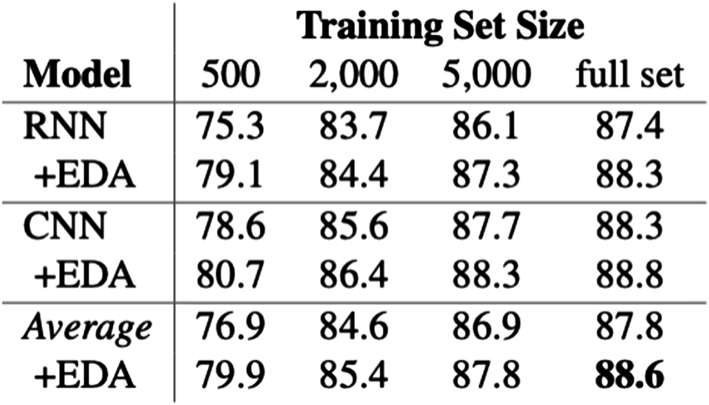
Success of EDA applied to 5 text classification datasets. A key takeaway from these results is the performance difference with less data. The gain is much more pronounced with 500 labeled examples, compared to 5,000 or the full training set

**Fig. 2:**
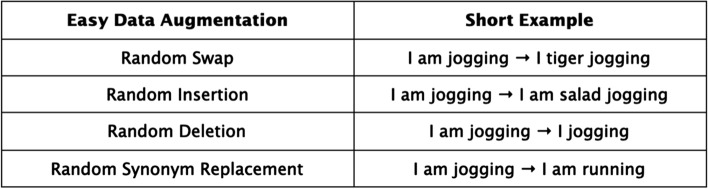
Examples of easy data augmentation transformations

Rule-based Augmentations construct rules to form augmented examples. This entails if-else programs for augmentation and symbolic templates to insert and re-arrange existing data. Easy Data Augmentation from Wei et al. [34] presents four augmentations. Figure 1 highlights the performance improvement with EDA, note the smallest subset of 500 labeled examples benefits the most. One of the main reasons to be excited about Easy Data Augmentation is that it is relatively easy to use off-the-shelf. Many of the Augmentations mentioned later in this survey, are still in the research phase, waiting for large-scale testing and adoption. Easy Data Augmentation includes random swapping, random deletion, random insertion, and random synonym replacement. Examples of this are shown in Fig. 2.

There are many opportunities to build on these augmentations. Firstly, we note that with random swapping, the classification of the word is incredibly useful. From the Data Augmentation perspective of introducing semantic invariances, “I am jogging”, is much more similar to “I am swimming” than “I am yelling”. Further designing token vocabularies with this kind of structure should lead to an improvement.

Programs for Rule-based augmentation further encompass many of the adversarial attacks that have been developed for NLP. Adversarial attacks are equivalent to augmentations, differing solely in the intention of their construction. As an example of a rule-based attack, Jin et al. [35] present TextFooler. TextFooler first computes word importance scores by looking at the change in output when deleting each word. TextFooler then selects the words which most significantly changed the outputs for synonym replacement. This is an example of a rule-based symbolic program that can be used to organize the construction of augmented examples.

Another rule-based strategy available is Regular Expression Augmentation. Regular Expression filtering is one of the most common ways to clean data that has been scraped from the internet, as well as several other data sources such as Clinical Notes [36]. Regular Expressions describe matching patterns in text. This is usually used to clean data, but it can also be used to find common forms of language and generate extensions that align with a graph-structured grammar. For example, matching patterns like “This object is adjective” and extending it with patterns such as, “and adjective”. Another strategy is to re-order the syntactics based on the grammar such as “This object is adjective” to “An adjective object”.

Min et al. [37] propose rules for augmentation based on syntactic heuristics. This includes Inversion, swapping the subject and object in sentences, and Passivization where the hypothesis in premise-hypothesis NLI (Natural-Language Inference) pairs are translated to the passive version of the sentence. An example of Inversion is the change from “The lawyer saw the actor” to “The actor saw the lawyer”. An example of Passivization is changing from “This small collection contains 16 El Grecos” to “This small collection is contained by 16 El Grecos”. The authors show improvement applying these augmentations on the HANS challenge set for NLI [38].

#### Graph-structured augmentation

An interesting opportunity for text data augmentation is to construct graph-structured representations of text data. This includes relation and entity encodings in knowledge graphs, grammatical structures in syntax trees, or metadata grounding language data, such as citation networks. These augmentations add explicit structural information, a relatively new integration with Deep Learning architectures. The addition of structure can aid in finding label-preserving transformations, representation analysis, and adding prior knowledge to the dataset or application. We will begin our analysis of Graph-Structured Augmentation by unpacking the difference between structured versus unstructured representations.

Deep Learning operates by converting high-dimensional, and sometimes sparse, data into lower-dimensional, continuous vector embedding spaces. The learned vector space has corresponding metrics such as L2 or cosine similarity distance functions. This is a core distinction from topological spaces, in which distance between points is not defined. A topological space is a more general mathematical space with less constraints than Euclidean or metric spaces. Topological spaces encode information that is challenging to integrate in modern Deep Learning architectures. Rather than designing entirely new architectures, we can leverage the power of structured data through the Data Augmentation interface.

One of the most utilized structures in language processing is the Knowledge Graph [39]. A Knowledge Graph is composed of (entity, relation, entity) tuple relations. The motivation of the augmentation scheme is that paths along the graph provide information about entities and relations which are challenging to represent without structure. Under the scope of Rule-based Augmentation, we presented the idea of synonym swap. One strategy to implement synonym swap would be to use a Knowledge Graph with “is equivalent” relationships to find synonyms. This can be more practical than manually defining dictionaries with synonym entries. This is especially the case thanks to rapid acceleration in automated knowledge graph construction from unlabeled data. Knowledge Graphs often contain more fine-grained relations as well.

Previously, we mentioned how random synonym replacement would benefit enormously from the perspective of preserving the class label with better swaps. Improved swaps describe transitions such as “I am jogging” to “I am running” compared to “I am yelling”, or even “I am market”. Structured language in graph-form is a very useful tool to achieve this augmentation capability. These kinds of graphs have been heavily developed with notable examples such as WordNet [40], Penn Treebank [41], and the ImageNet class label structure [17]. Graphs such as WordNet describe words in relationship to one another through “synsets”.

Graphs are made up of nodes and edges. In WordNet, each node represents a word such as “tiger”. The genius of WordNet is the simplification of which edges to connect. In WordNet, the nodes are connected with the same edge type, a “synset” relationship. Synsets are loosely defined as words belonging to a similar semantic category. The word “tiger” would have a synset relation with nodes such as “lion” or “jaguar”. The word “tiger” may also have finer-grained synset relations with nodes that describe more particular types of tigers. WordNet is an example of a Graph-Structured Augmentation that builds on synonym replacement. WordNet describes a graph where each node is related to another graph by being a “synset”.

We additionally consider graphs that contain finer grained edge classifications, this kind of graph is frequently referred to as a Knowledge Graph [39]. As an example, CoV-KGE [42] contains 39 different types of edges relating biomedical concept nodes such as drugs or potential binding targets. Huang et al. [43] provide another interesting example of constructing a knowledge graph from the long context provided as input to abstractive summarization. This graph enables semantic swaps that preserve global consistency.

Another heavily studied area of adding structure to text data is known as syntactic parsing. Syntactic parsing describes different tasks that require structural analysis of text such as the construction of syntax or dependency trees. Recently, Glavas and Vulic [44] demonstrated that supervised syntactic parsing offered little to no benefit in the modern pre-train, then fine-tune pipeline with large language models.

The final use of structure for Text Data Augmentation we consider is to integrate metadata via structural information. For example, scientific literature mining has become a very popular application of NLP. These applications could benefit from the underlying citation network characterizing these papers, in addition to the text content of the papers themselves. Particularly, network structure has played an enormous role in biology and medicine. Li et al. [45] present many of these graphs in high-level application domains such as molecules, genomics, therapeutics, and healthcare. The integration of this structure with text data could be a key component to grounding text representations.

In the theme of our survey, we note that these auxiliary graphs may benefit from augmentation as well. Data Augmentation for explicitly graph-structured data is still in its early stages. Zhao et al. [46] propose an edge augmentation technique that “exposes GNNs to likely (but nonexistent) edges and limiting exposure to unlikely (but existent) ones” [46]. This graph augmentation leads to an average accuracy improvement of 5% across 6 popular node classification datasets. Kong et al. [47] further demonstrate the effectiveness of adversarially controlled node feature augmentation on graph classification.

In the section, Practical Considerations for Implementation, we will present the use of consistency regularization and contrastive learning to further enforce the use of augmented data in training. Building on these ideas, we can use graph-structures to assign nearest neighbor assignments and regularize embeddings. Neural Structured Learning [48] describes constructing a graph connecting instances that share fine-grained class labels. This is used to penalize a misclassification of “golden retriever” less so than “elephant” if the ground truth label is “labrador retriever”. Li et al. [49] similarly construct an embedding graph to enforce consistency between predictions of strong and weakly augmented data.

#### MixUp augmentation

MixUp Augmentation describes forming new examples by meshing existing examples together, sometimes blending the labels as well. As an example, MixUp may take half of one text sequence and concatenate it with half of another sequence in the dataset to form a new example. MixUp may be one of the best interfaces available to connect distant points and illuminate a path of interpolation.

**Fig. 3:**
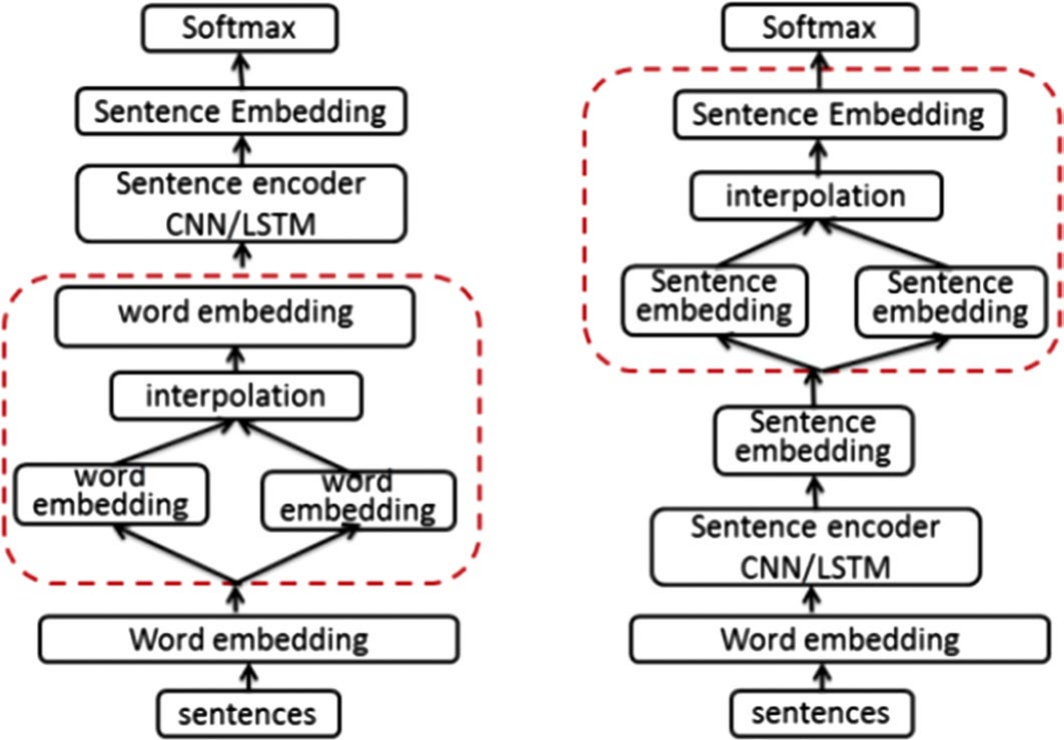
Left, word-level mixup. Right, sentence-level mixup. The red outline highlights where augmentation occurs in the processing pipeline

Most implementations of MixUp vary with respect to the layer in which samples are interpolated. Guo et al. [50] test MixUp at word and sentence levels. This difference is shown in Fig. 3. Their wordMixup technique combines existing samples by averaging embedding vectors at the input layer. The sentMixup approach combines existing samples by averaging sentence embeddings as each original sequence is passed through siamese encoders. Their experiments find a significant improvement in reducing overfitting compared to no regularization or using dropout.

#### Feature space augmentation

Feature Space Augmentation describes augmenting data in the intermediate representation space of Deep Neural Networks. Nearly all Deep Neural Networks follow a sequential processing structure where input data is progressively transformed into distributed representations and eventually, task-specific predictions. Feature Space Augmentations isolate intermediate features and apply noise to form new data instances. This noise could be sampled from standard uniform or gaussian distributions, or they could be designed with adversarial controllers.

**Fig. 4:**
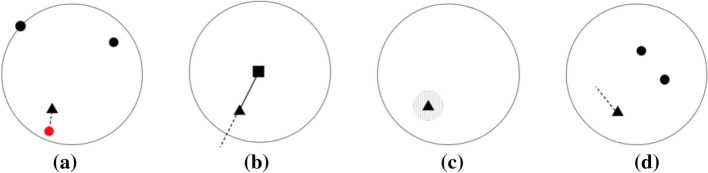
Directions for feature space augmentation explored in MODALS

MODALS [51] presents a few strategies for feature space augmentations. Shown in Fig. 4, these strategies describe how to move along class boundaries to form new examples in the feature space. Hard example interpolation (a) forms a new example by moving it in the direction of existing embeddings that lie on the decision boundary for classification. Hard example extrapolation (b) describes moving existing examples along the same angle they currently lie from the mean vector of the class boundary. Gaussian noise (c) entails adding Gaussian noise in the feature space. Difference transform (d) moves an existing sample in the directional distance calculated from two separate points in the same class. As described as one of the general Motifs Of Data Augmentation, MODALS aims to strengthen decision boundaries. Research in Supervised Contrastive Learning [52], replacing the commonly used KL-divergence of logits and class labels with contrastive losses such as NCE with positives and negatives formed based on class labels, has been shown to improve these boundaries. It could be useful to explore how this benefits the MODALS algorithm.

We also consider Differentiable Data Augmentation [53] techniques to fall under the umbrella of Feature Space Augmentation. Data Augmentation is a function f(x) that produces augmented examples x’. Similar to any other layers in the network, we can treat the beginning of the network as an augmentation module and backpropagate gradients through it. We can also separate the augmentation function and add it to the inputs such that the transformation is not too dramatic, akin to adding an optimized noise map to the input. Minderer et al. [54] use this technique to facilitate self-supervised pretext tasks.

### Neural augmentation

The following augmentations rely on auxiliary neural networks to generate new training data. This entails using a model trained on supervised Neural Machine Translation datasets to translate from one language to another and back to sample new instances, or a model trained on generative language modeling to replace masked out tokens or sentences to produce new data. We additionally discuss the use of neural style transfer in NLP to translate from one writing style to another or one semantic characteristic such as formal to casual writing.

#### Back-translation augmentation

Back-translation describes translating text from one language to another and then back from the translation to the original language. An example could be taking 1,000 IMDB movie reviews in English and translating them to French and back, Chinese and back, or Arabian and back. There has been an enormous interest in machine translation. This has resulted in the curation of large labeled datasets of parallel sentences. We can also imagine the use of other text datasets such as translations between programming languages or writing styles as we describe in more detail under Style Augmentation.

Back-translation leverages the semantic invariances encoded in supervised translation datasets to produce semantic invariances for the sake of augmentation. Also interestingly, back-translation is used to train unsupervised translation models by enforcing consistency on the back-translations. This form of back-translation is also heavily used to train machine translation models with a large set of monolingual data and a limited set of paired translation data. Outside of translation we could imagine structuring these domain pairings such as scientific papers and news articles or college-level and high-level reading and so on.

An interesting design question with this may be to weigh the importance of using a high performance machine translation model for the back-translation. However, as stated by Pham et al., the lesson has been “better translation quality of the pseudo-parallel data does not necessarily lead to a better final translation model, while lower-quality but diverse data often yields stronger results instead” [55]. The curation of paired languages and domains could also impact the final performance. Exploring back-translation augmentation for question answering Longpre et al. discuss “curating our input data and learning regime to encourage representations that are not biased by any one domain or distribution” [56].

#### Style augmentation

Finally, we present another augmentation strategy utilizing Deep Networks to augment data for the training of other Deep Nets. In our previous survey of Image Data Augmentation, we explored works that use Neural Style Transfer for augmentation. Artistic style transfers such as a picasso-themed dog image, may be useful as an OOD augmentation in a Negative Data Augmentation framework, which we will present later. However, we are more interested in styles within the dataset. This is an interesting strategy to prevent overfitting to high-frequency features or blurring out the form of language such as to focus on meaning. In the text data domain, this could describe transferring the writing-style of one author to another for applications such as abstractive summarization or context for extractive question answering.

Data Augmentation is often deployed to focus models on semantics, rather than particular forms of language. These particular forms could emerge from one author’s writing style or general tonality in the language such as an optimistic versus a pessimistic writer. Style transfer offers an interesting window to extract semantic similarities between writing styles. This could help with modeling contexts in question answering systems or documents for information retrieval.

#### Generative data augmentation

Generative Data Augmentation is one of the most exciting emerging ideas in Deep Learning. This includes generating photorealistic facial images [57] or indistinguishable text passages [14]. These models have been very useful for Transfer Learning, but the question remains: What is the killer application of the generative task? These generations are certainly interesting for artistic applications, but more importantly is their use for representation learning and Data Augmentation.

We note a core distinction in the use of generative models for Data Augmentation. A popular use is to take a pre-trained language model of the shelf and optionally fine-tune it further with the language modeling task. This is the standard operating procedure of Transfer Learning. However, the fine-tuning is usually done with the Supervised Learning task, rather than additional language modeling. The pre-trained language models have learned many interesting properties of language because they are trained on massive datasets. An interesting example that is publicly available is The Pile [58]. The Pile is 800GB of text data spanning Wikipedia, comment forums, entire books, and many more examples of data like this. Even though these models and datasets are very impressive, additional benefits will likely be achieved by domain-tuning with additional language modeling on the limited dataset.

Language modeling is a very useful pre-training stage and we often have more data for language modeling than a downstream task like question-answering. Whereas we may only have 100 question-answer pairs, the question, answer, and surrounding context could easily contain 300 words each, accounting for a total of 3,000 words for constructing language modeling examples. A dataset size of 3,000 compared to 100 can make a large difference in success with Deep Learning and is the prime reason for our interest in Data Augmentation to begin with. Gururangan et al. [59] present an argument for this use of language models since downstream performance is dramatically improved when pre-training on a relevant dataset. This distinction of “relevant dataset” is in contrasting reference to what is used to train models like GPT-3 [14].

One of the most popular strategies for training a language model for Generative Data Augmentation is Conditional BERT (C-BERT) [60]. C-BERT augments data by replacing masked out tokens of the original instance. The key novelty is that it takes an embedding of the class label as input, such as to preserve the semantic label when replacing masked out tokens. This targets the label-preserving property of Data Augmentation. The C-BERT training strategy can be used when fine-tuning a model pre-trained on another dataset or starting from a random initialization.

An emerging strategy to adapt pre-trained generative models to downstream tasks is to re-purpose the interface of masking out tokens. This is known as prompting. The output of language models can be guided with text templates for the sake of generating or labeling new data. Testing the efficacy of prompting with respect to the objective of learning from limited data, Scao and Rush [61] show that prompting is often worth 100s of data points on SuperGLUE classification tasks [32]. This is in direct comparison with the more heavily studied paradigm of Transfer Learning, head-based fine-tuning. We will present a few variants on implementing prompts, this includes in-context learning, pattern-exploiting training, and prompt tuning.

The first implementation of prompting we consider is in-context learning. In-context learning became well known when demonstrated with GPT-3. The idea is to prepend each input with a fixed task description and a collection of examples of the task. This does not require any further gradient updates of the model. Brown et al. [14] show that scale is crucial to making this work reporting significant performance drops from 175B parameters to 13B and less. This technique has likely not yet hit its ceiling, especially with the development of transformer models that can in sequences longer than 512 tokens as inputs. Similar to excitement about retrieval-augmented modeling, this will allow in-context learning models to process more demonstrations of the task. However, due to limitations of scale, methods that continue with gradient updates are more practically useful.

The next implementation of prompting we will present is prompt tuning. Prompt tuning describes first embedding the prompt into a continuous space, and then optimizing the embedding with gradient descent while keeping the rest of the network frozen. Similarly to GPT-3, Lester et al. [62] show that scale improves performance with prompt tuning and that prompt tuning significantly outperforms the in-context learning results reported from Brown et al. [14]. Performance can be further improved by ensembling optimized prompts and running inference as a single batch of the input and the appended prompts. Tuned prompt ensembling improves the average performance of the prompts on SuperGLUE from 88.5, and the best performing individual prompt at 89.8, to 90.5. The authors further highlight that analysis of the optimized prompt embedding can aid in task complexity and similarity metrics, as well as Meta-Learning. Prompt tuning shares the same underlying concept of prepending context to the input of downstream tasks to facilitate fine-tuning, however this technique is more in line with research on Transfer Learning with minimal modifications. For example, adapter layers [63] aim to introduce a small number of parameters to fine-tune a pre-trained Transformer.

An emerging theme in the pre-train then fine-tune paradigm has been that domain and task alignment tends to improve fine-tuned performance. Gururangan et al. [59] demonstrate the effectiveness of data domain alignment and Zhang et al. [64] demonstrate effectiveness of task alignment in the proposed PEGASUS algorithm. In correspondence with the lesson of alignment, Zhong et al. [65] tune language models to be better fitted to answer prompts. This is done by manually annotating 441 questions across 43 existing datasets that map every task to a “Yes” or “No” answer. Measured by AUC-ROC plots, the authors show that further fine-tuning on prompt specialization improves these models and that this also benefits from scale. The authors call for the organization of NLP datasets into unified formats that better aids in fine-tuning models for answering prompts.

Pattern exploiting training (PET) [66] uses the pre-trained language model to label task-specific unlabeled data. This is done with manually-defined templates that convert the supervised learning task into a language modeling task. The outputs of the language model are then mapped to supervised learning labels with a verbalizer. Gradient-descent optimization is applied to verbalized outputs to fine-tune it with the same cross-entropy loss function used to train classifiers. Schick and Shutze [67] demonstrated that the PET technique enables much smaller models to surpass GPT-3 with 32 labeled examples from SuperGLUE. Tam et al. [68] further developed the algorithm to ADAPET. ADAPET utilizes dense supervision in the labeling task, applying the loss to the entire vocabulary distribution without a verbalizer and additional requiring the model to predict the masked tokens in the context given the label, similarly to conditional-BERT. ADAPET outperforms PET without the use of task-specific unlabeled data.

A limitation to pattern-exploiting training, in-context learning, and prompt tuning, is that they require retaining a large language model for downstream tasks. Most applications are interested in compressing these models for the sake of efficiency. Under the scope of Label Augmentation, we will present the use of knowledge distillation. For now, we consider compression by generating data to train a smaller model with. This approach is most similar to pattern-exploiting training, except that rather than use the pre-trained language model to label data, we will instead use it to generate entire examples.

Drawing inspiration from the success of MixUp, which was presented in further detail in MixUp Augmentation, Yoo et al. developed GPT3Mix [69]. The input to GPT3Mix begins with a Task Specification that defines the task such as, “Text Type T = movie review, Label Type L = sentiment”. Akin to MixUp, the next inputs are examples of the task formulated as “text type: example text k (label type: example label k)”, such as “Example 1: The cat is running my mat. (negative)”. The final piece of the input is the template to generate new examples. Further, the generated example is “soft-labeled” by the generating probabilities of each token in the process of generating the new example. GPT3Mix achieves massive performance improvements over no augmentation, Easy Data Augmentation, and BackTranslation when subsetting available data to extreme levels such as 0.1% and 0.3%.

Schick and Shutze [26] also explore the strategy of generating data from language models, presenting Datsets from Instructions (DINO). DINO uses a task description and one example from the dataset to generate pairwise classification datasets. Interestingly, they contrast task descriptions which entail the resulting label to decode language model generation. For example, the task description could begin with “Write two sentences that” and continue with either “mean the same thing” or “are on completely different topics”. The generation accounts for the token another label description would generate. Evaluated on the STS text similarity dataset, representations learned from DINO show improvements over state-of-the-art sentence embedding techniques trained with supervised learning, such as Universal Sentence Encoders [70] and Siamese BERT and RoBERTa models [71].

While built on the same underlying concept, discrete versus continuous prompt search diverge heavily from one another. Discrete prompt search has the benefit of interpretability. For example, comparing different task descriptions and examples provided by a human annotator offers insights into what the model has learned. However, prompt optimization in the continuous embedding space fully automates the search. Continuous prompt optimization is likely more susceptible to overfitting due to the freedom of the optimization space.

Another somewhat similar theme to prompting in NLP has been to augment knowledge-enhanced text generation with retrieval. Popular models include Retrieval-Augmented Generation (RAG) [72], and Retrieval-Augmented Language Model Pre-training (REALM) [73]. Shuster et al. [74] show how retrieving information to prepend to the input reduces the problem of hallucination in text generation. Once this retrieved information is embedded into the continuous representation space of language models, it is a similar optimization problem as prompt tuning.

Another interesting idea is the intersection of Data Privacy and Generative Data Augmentation. Can we store data in the parameters of models instead of centralized databases? The idea of Federated Learning [75] is to send copies of the global model weights to a local database such as to avoid a centralized database. Which models should we send to local databases? Classifiers or generative models? If we send a generative model, we have the potential to cover more of the data distribution and learn more about general data manifolds such as the use of language more broadly, however, we risk exposing more critical information [76].

### Label augmentation

Supervised Learning, describes fitting an input, x, to a label, y. Throughout this survey, we have presented strategies for regularizing the x values. In this section, we explore research looking to entertain the y class labels. The most successful example of this is Knowledge Distillation [77]. Knowledge Distillation describes transforming the traditional one-hot encoded y labels into a soft distribution by re-labeling xs with the logits of another neural network’s prediction. This has been very influential in compression such as DistilBERT [78], information retrieval [79], and achieving state-of-the-art classification results in Computer Vision [80].

In addition to Knowledge Distillation, several other strategies have been developed to augment the label space. Label smoothing uses a heuristic adjustment to the density on negative classes and has been highly influential for training classifiers [81] and generative adversarial networks [82]. Another exciting approach is the use of a meta-controller, similar to knowledge distillation, but massively different in that the Teacher is learning from the gradients of the Student’s loss to update the label augmentation. Notable examples exploring this include Meta Pseudo Labels [83] and Teaching with Commentaries [84]. This ambitious idea of learning to augment data through outer-inner loop gradients have also been explored in the data space, x, with Generative Teaching Networks [12]. As of the time of this writing, Generative Teaching Networks have only been applied to image data. A similar idea is “Meta Back-Translation” [55], in this work, the authors “propose a meta-learning framework where the back-translation model learns to match the forward translation model’s gradients on the development data with those on the pseudo-parallel data.”

Thakur et al. [85] present the Augmented SBERT to augment data labels for distillation. The authors note that the cross-encoder, although much slower and less efficient than bi-encoders, tends to reach higher accuracy on pairwise classification tasks such as ranking or duplicate question detection. The paper proposes to label data with the cross-encoder and fit these augmented labels with the bi-encoder. Also worth mentioning is that the cross-encoder heavily outperforms the bi-encoder with less training data. Thakur et al. find a significant benefit strategically selecting data to soft label with the cross encoder. We have found this idea throughout experiments in Data Augmentation, discussing it further in our Discussion section under Curriculum Learning.

## Testing generalization with data augmentation

The holy grail of Machine Learning is to achieve out-of-distribution (OOD) generalization. This is distinct from in-distribution generalization where the training and test sets are sampled from the same data distribution. In order to measure OOD generalization, we need to make assumptions about how the distribution will shift. As Arjvosky writes, “if the test data is arbitrary or unrelated to the training data, then generalization is obviously futile” [86]. Chollet further describes the relationship between system-centric and developer-aware generalization, as well as levels of generalization such as absent, local, broad, and extreme [87]. We argue that Data Augmentation is the natural interface to quantify the relationship between test and train data distributions and levels of generalization.

**Fig. 5:**
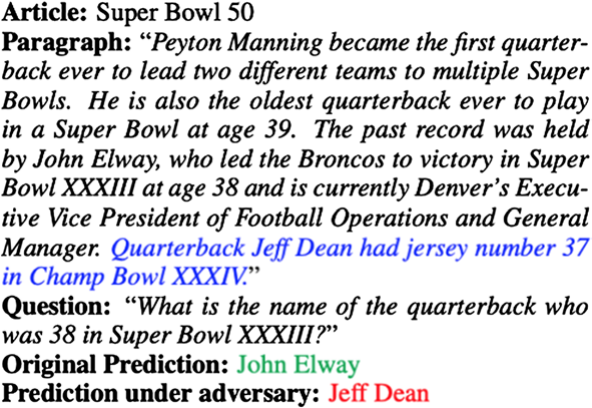
Fooled by injected text. Image taken from Jia and Liang [89]

A classic tool to test for generalization is to simply report the difference in accuracy between the training and test sets. However, as shown in papers such as Deep Double Descent [88], the phenomenon of overfitting is generally poorly understood with large-scale Deep Neural Networks. We believe it is more practical to study overfitting and generalization in the data space. For example, the success of adversarial examples shows that Deep Neural Networks cannot generalize to distributions added with adversarially optimized noise maps. Jia and Liang [89] show that models trained on SQuAD cannot generalize when adversarially optimized sentences are added to the context, an example of this is shown in Fig. 5. In addition to adversarial attacks, many other datasets show intuitive examples of distribution shifts where Deep Neural Networks fail to generalize.

We present Data Augmentation as a black-box test for generalization. CheckList [90] proposes a foundational idea for these kinds of tests in NLP. CheckList is designed to test the linguistic capabilities of models such as robustness to negation, vocabulary perturbations, or temporal consistency. We view this as introducing a distribution shift of linguistic phenomena in the test set. Clark et al. [91] construct a toy example for transformers to see how far they can generalize fact chaining. In this test, the training data requires the model to chain together more or less facts than are tested in the test set. Again, the distribution shift is controlled with an intuitive interface again to Data Augmentation. Finally, WILDS [92] is a collection of real-world distribution shifts. These real-world shifts can also be mapped to Data Augmentations.

Kaushiik et al. [24] describes employing human-labelers to construct a set of counterfactual movie reviews and natural language inference examples. The authors construct an elegant annotation interface and task Mechanical Turk workers to minimally edit examples such as to switch the label. For example, converting “The world of Atlantis, hidden beneath the earth’s core, is fantastic” to “The world of Atlantis, hidden beneath the earth’s core is supposed to be fantastic”. For movie reviews, the authors group the workers’ revisions into categories such as recasting fact as hoped for, suggesting sarcasm, inserting modifiers, inserting phrases, diminishing value qualifiers, differing perspectives, and changing ratings. For natural language inference, the authors group the workers’ revisions into categories such as modifying/removing actions, substituting entities, adding details to entities, inserting relationships, numerical modifications, using/removing negation, and unrelated hypothesis. These examples are constructed for testing generalization to these counterfactual examples.

Returning to our description of Generative Data Augmentation, are generative models capable of making these edits? If GPT-3 was given an IMDB review with the task prompt of “change this movie review from positive to negative”, it could probably manage it. We leave it to future work to investigate the generalization shifts induced by human-designed counterfactuals and generative models. To further motivate this study, the authors note that their dataset construction came with a hefty price tag of $10,778.14. Inference costs of generative models are unlikely to approach this cost, unless working with extremely large models. Highlighting that a similar categorization of the changes as Kaushik et al. use [24] could help us understand the linguistic phenomena underlying this kind of generalization test.

Generative Data Augmentation provides another lens to study generalization. Nakkiran et al. propose a novel way of studying generalization in “The Deep Bootstrap Framework” [93]. The idea is to compare the Online test error to the Bootstrap test error. The Online error describes the performance of a model trained on an infinite data stream, i.e. without repeating samples. The Bootstrap test error describes the common training setup in Deep Learning, repeating batches of the same data. The authors simulate the Online learning scenario by fitting a generative model, in this particular case a Denoising diffusion probabilistic model [94]. The generative model is used to sample 6 million examples, compared to the standard 50,000 samples used to train CIFAR-10. Garg et al. [95] additionally propose RATT, a technique that analyzes learning curves and generalization when randomly labeled unlabeled data is added to the training batch. The augmentations described in this survey may be able to simulate this unlabeled data and provide similar insights.

To conclude, when is overfitting problematic? How much of a data distribution are modern neural networks capable of covering? Deep Neural Networks have a remarkable ability to interpolate within the training data distribution. A potential solution could be to leverage Data Augmentation to expand the training distribution such that there are no reasonable out-of-distribution shifts in the test sets. Even if all the potential distributions cannot be compressed into a single neural network, this interface can illuminate where the model will fail.

## Image versus text augmentation

Our survey on Text Data Augmentation for Deep Learning is intended to follow a similar format as our prior work on Image Data Augmentation for Deep Learning [6]. We note there are many similarities between the Easy Data Augmentations and basic geometric and color space transformations used in Computer Vision. Most similarly, both are easy to implement and complement nearly any problem working with text or image data respectively. We have described how Easy Data Augmentation can easily interface with text classification, pairwise classification, extractive question answering, abstractive summarization, and chatbots, to name a few. Similarly, geometric and color space transformations in Computer Vision are used in image classification, object detection, semantic segmentation, and image generation.

As described in the beginning of our survey, Data Augmentation biases the model towards certain semantic invariances. Image Data Augmentation has largely been successful because it is easy to think semantic invariances relevant to vision. These include semantic invariance to horizontal flips, rotations, and increased brightness, to name a few. Comparatively, it is much harder to define transformations to text data that are guaranteed to be semantically invariant. All of the augmentations described in Easy Data Augmentation have the potential to perturb the original data such that it changes the ground truth label, y.

Another interesting trend is the integration of vision and language in recent models such as CLIP and DALL-E. For the sake of Data Augmentation, a notable example is Vokenization from Tan and Bansal [96]. The authors align tokens such as “humans” with images of “humans” and so on, even for verbs such as “speaking”. The masked language modeling task then uses the visual tokens as additional supervision for predicting masked out tokens. There is some noise in this alignment such as finding a visual token for words such as “by” or “the”. Tan and Basil report visual grounding ratios for tokens of 54.8%, 57.6%, and 41.7% on curated vision-language datasets compared to 26.6%, 27.7%, and 28.3% for solely language corpora. Across the SST-2, QNLI, QQP, MNLI, SQuAD v1.1 and v2.0, and SWAG benchmark tasks, Vokenization improves BERT-Large from 79.4 to 82.1 and RoBERTa-Large from 77.6 to 80.6. There are many interesting vision-language datasets labeled for tasks such as visual question answering, image captioning, and text-image retrieval, to name a few. Vision-language Data Augmentation schemes such as Vokenization look to be a very promising area of research.

A recent trend in Image Data Augmentation has been its integration in the training of generative models, namely generative adversarial networks (GANs) [97]. The GAN framework, similar to the ELECTRA model [98], consists of a generator and a discriminator. The generator transforms random noise into images and the discriminator classifies these images as either coming from the generator or the provided training set. Following, we will describe why this does not work as well as autoregressive modeling for text. Returning to how Data Augmentation has been used for GANs, this investigation began with Zhang et al.’s work on consistency regularization [99]. Consistency regularization requires the discriminator to make the same classification on a real image and an augmented view of that same image. Unfortunately, this led to the augmentations being “leaked” into the generated distribution such that the generator produces augmented data as well.

We will end this discussion by presenting some ideas from LeCun and Misra [100] on the key distinction between generative modeling between Images and Text. The key issue stated in the article is handling uncertainty. As an example, take the masked token completion task: “The mask chases the mask in the savana”. LeCun and Misra point out that the language model can easily “associate a score or a probability to all words in the vocabulary: high score for lion’, ‘cheetah’, and a few other predators, and low scores for all other words in the vocabulary” [100]. In comparison, applying this kind of density on candidate images in highly intractable. The missing token can only be 1 of a typical 30,000 tokens, whereas a missing 8x8 RGB patch can take on a ridiculously large, 255x8x8x3 values. Therefore, image models need to rely on energy-based models that learn joint embedding spaces and assign similarity scores, rather than exactly modeling the probability of each missing patch. Perhaps the GAN framework, or something similar, will take over in NLP once generative modeling expands its scope to sentence-level or paragraph-level generation, such as the pre-training task used for abstractive summarization in PEGASUS [64].

Another interesting success of Data Augmentation has been its application in Reinforcement Learning. This has been heavily studied with Robotic Control from Visual Inputs and the Atari benchmark. One of the biggest bottlenecks with robotic learning, and most deep reinforcement learning problems, is a lack of data. It is challenging to restart a robot laundry folder back to the beginning of the unfolded shirt and collect millions of trajectories. To solve this problem, researchers have turned to forming augmented trajectories from collections in a replay buffer. Amongst many applications of reinforcement learning with Text data that have been proposed, patient care control is particularly exciting. Ji et al. [101] explore the use of model-based reinforcement learning for patient care of septic patients using the MIMIC-III dataset [102]. The authors use clinical notes to sanity check the model-based rollouts of physiological patient state markers. A promising area of research will be to apply Text Data Augmentation to collected clinical note trajectories to improve patient care and trajectory simulation.

## Practical considerations for implementation

This section presents many details of implementing Text Data Augmentation that make a large performance difference in terms of evaluation metrics and training efficiency.

### Consistency regularization

Consistency regularization is a strong compliment to the priors introduced via Data Augmentation. A consistency loss requires a model to minimize the distance in representations of an instance and the augmented example derived from it. In line with the motif of strengthening decision boundaries, consistency regularization enforces a connection between original and augmented samples. This is usually implemented in a multi-task learning framework where a model simultaneously optimizes the downstream task and a secondary consistency term.

Consistency regularization has been successfully applied to translate between programming languages by enforcing consistency on back-translations [103]. Alberti et al. [104] use a slightly different form of consistency regularization to generate synthetic question-answer pairs. Rather than minimizing the distance between representations of original and augmented examples, the framework requires that the model outputs the exact same answer when predicting from context, question inputs as when a separate model generates the question from context, answer inputs. The original BERT-Large model achieves an F1 score of 83.1 when fine-tuned on the SQuAD2. Fine-tuning BERT with an additional 7 million questions generated with the consistency condition improves performance to 84.8.

**Fig. 6:**
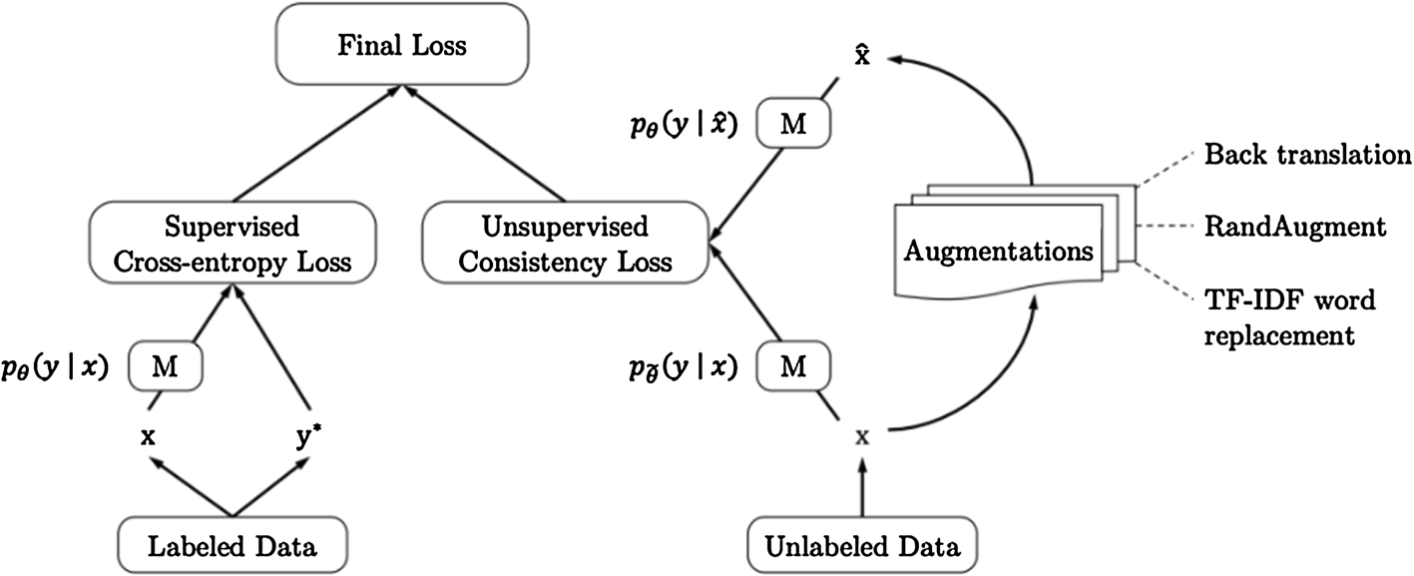
Unsupervised data augmentation schema. Image taken from Xie et al. [105]

Consistency regularization is a common technique for self-supervised representation learning because unlabeled data should still have this property of consistent representations before and after augmentation. Xie et al. [105] deploy consistency regularization as shown in Fig. 6. This technique surpasses the previous state-of-the-arts trained solely with supervised learning using significantly less data. These improvements continue even in the extreme case of only 20 labeled examples. As an example of the performance gain, the fine-tuned BERT model achieves a 6.5% error rate on IMDB review classification, which is reduced to 4.2% with UDA. The multi-task loss formulation is also fairly common in consistency regularization implementations.

### Contrastive learning

Contrastive learning differs from consistency regularization by utilizing negative samples to normalize the loss function. This is a critical distinction because the negative samples can provide a significant learning signal. We believe that the development of Text Data Augmentation can benefit from adapting successful examples in Computer Vision. The use of Data Augmentation to power contrastive self-supervised learning has been one of the most interesting stories in Computer Vision. This involves frameworks such as SimCLR [106], MoCo [107], SwAV [108], and BYOL [109], to name a few. This training strategy should be well suited for information retrieval in NLP.

Krishna et al. [110] propose contrastive REALM (c-REALM). The contrastive loss is used to align the embedding of the question and supervised answer, and contrast the question with other supervised answers from the mini-batch. However, this technique of contrastive learning is more akin to supervised contrastive learning [52], than frameworks such as SimCLR. In SimCLR, Data Augmentation is used to form the positive pairs. This strategy has not been heavily explored in information retrieval, likely due to the lack of augmentations. Hopefully, the list we have provided will help those interested pursue this idea.

Gunel et al. [111] demonstrate significant improvements on GLUE benchmark tasks by training with a supervised contrastive loss in addition to cross-entropy loss on one-hot encoded label vectors. The gain is especially pronounced when learning from 20 labeled examples, while they do not report much of a difference at 1,000 labeled examples. In addition to quantitative metrics, the authors highlight that the embeddings of classes are much more spread out through the lens of a t-SNE visualization.

Contrastive learning, similarly to consistency regularization, describes making the representation of an instance and a transformation-derived pair similar. However, contrastive learning adds a negative normalization that additionally pushes these representations away from other instances in the samples mini-batch. Contrastive learning has achieved large advances in representation Computer Vision such as SimCLR [106] and MoCo [107]. Using Data Augmentation for contrastive learning is a very promising area of research with recent extensions to the information-retrieval language model REALM [73]. We refer interested readers to a report from Rethmeier and Augenstein [112] for more details on early efforts to apply contrastive learning to NLP.

Consistency regularization and contrastive learning are candidate solutions to a common problem found by inspecting model performance. For example, Thorne et al. [113] find that fact verification models achieve better accuracy when classifying if claims are supported or refuted by the evidence when ignoring the evidence. Contrastive learning would require the model to correctly associated supporting evidence by contrasting it with refuting evidence. Consistency Regularization would more so describe having a similar prediction when the evidence has been slightly perturbed, such as inserting a random word or replacing it with a paraphrase that shares the same semantics.

### Negative data augmentation

Negative Data Augmentation is a similar concept to the negative examples used in contrastive learning. However, a key difference is that contrastive learning generally uses other data points as the negatives, whereas Negative Data Augmentation entails applying aggressive augmentations. These augmentations are not just limited to label corruptions, but may push the example out of the natural language distribution entirely. Returning to the motif of Meaning versus Form [28] these augmentations may not be useful for learning meaning, but they can help reinforce the form of natural language. Sinha et al. [114] demonstrate how this can be used to improve contrastive learning and generative adversarial networks.

### Augmentation controllers

A large contributor to the success of Data Augmentation in Computer Vision is the development of controllers. Controllers reference algorithms that optimize the strength of augmentations throughout training. The strength of augmentations describe the magnitude of operation such as inserting 3 additional words compared to 15. Augmentation strength also describes how many augmentations are stacked together such as random insertion followed by deletion followed by back-translation and so on, described more next. Successful controllers such as AutoAugment [7], Population-Based Augmentation [8], or RandAugment [9] have not yet seen large-scale adoption in NLP.

When applying Easy Data Augmentation, several hyperparameters arise. Hyperparameter optimization is one of the active areas of Deep Learning research [115–117]. This presents a perfect problem to find optimal values for random augmentation samplings, as well as magnitudes such as: how many tokens to delete? SpanBERT [118], for example, shows that instead of masking out single tokens for language modeling, masking out multiple tokens at a time, known as spans, results in better downstream performance.

### Adversarial augmentation

Adversarial attacks and the use of adversarially optimized inputs for augmentation is very similar to the previous discussion on controllers. The key differentiation is that adversarially controllers target misclassifications whereas controllers generally try to avoid misclassifications. Particularly, adversarial optimization aims to improve robustness to high-frequency pattern shifts. Adversarial attacks on text data generally range from introducing typos to swiping out individual or chunks of words. There is a great deal of ambiguity with this since many of these perturbations would be cleaned and filtered by the text data preprocessing techniques such as spell checkers, case normalizations, or regular expression filtering.

**Fig. 7:**
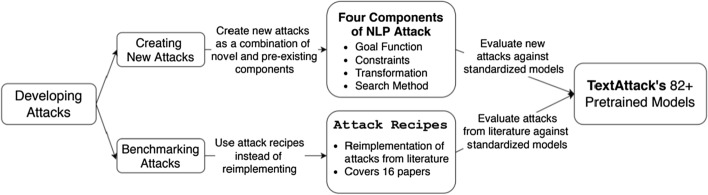
Developing attacks in TextAttack [119]

TextAttack [119] is an open-source library implementing adversarial text attacks and providing APIs for Data Augmentation. There are four main components of an attack in the TextAttack framework, a goal function, constraints, transformations, and a search method. This pipeline is illustrated in Fig. 7. The goal function defines the target output, for example instead of solely flipping the predicted output we may want to target a 50-50 density. The constraints define how far the input can be changed. The transformation describes the tools available to change the input such as synonym swaps, deletions, applying back-translation, and all the other techniques discussed previously. Finally, the search method describes the algorithm for searching for the attack. Similar to our discussion of controllers there are many different ways to perform black-box searches such as grid or random searches, bayesian optimization, and evolutionary search, to name a few [115].

A key consideration with adversarial augmentation is how quickly we can construct adversarial examples. Many adversarial example construction techniques such as Szegedy et al. [120] rely on iterative optimization such as L-BFGS to find the adversarial example. This would be a significant bottleneck in Deep Learning training to wait for the adversarial search at each training batch. Towards solving this issue, Wang et al. [121] reduce time consumption up to 60% with their DEAT algorithm. The high-level idea of DEAT is to use batch replay to avoid repeatedly computing adversarial batches.

### Stacking augmentations

Stacking augmentations is a strategy that has improved vision models but is less straightforward to apply to text data. One strategy for this is CoDA [122]. CoDA introduces a local consistency loss to make sure stacking augmentations has not overly corrupted the sample, and a global loss to preserve local neighborhoods around the original instance.

### Tokenization

The preprocessing pipeline of tokenization presents a formidable challenge for implementing Data Augmentations. It is common to tokenize, or convert word tokens to their respect numeric index in a vocabulary-embedding lookup table offline before it reaches the Data Loader itself. Applying Data Augmentations on these index lists could require significantly more engineering effort. Even for simple synonym replacement, additional code will have to be written to construct dictionaries of the synonyms index value for swaps. Notably, researchers are exploring tokenizer-free models such as byT5 [123] and CANINE [124]. These models process byte-level sequences such as ASCII codes [125, 126] and will require special processing to integrate these augmentations.

### Position embeddings

Another more subtle detail of Transformer implementations are the use of position embeddings. The original Transformer [92] uses sine and cosine functions to integrate positional information into text sequences. Another subtle Data Augmentation could be to explore perturbing the parameters that render these encodings.

### Augmentation on CPUs or GPUs?

Another important aspect of Data Augmentation is to understand the typical data preprocessing pipeline from CPUs to GPUs. It has been standard practice to apply Data Augmentation to data on the CPU before it is passed to the GPU for model training. However, recent practice has looked at applying Data Augmentation directly on the GPU. This is done in Keras, for example, by adding Data Augmentation as a layer in the model immediately after the input layer. It is also worth noting clever schemes such as Data Echoing from Choi et al. [127] that apply additional techniques to avoid idle time between CPU data loading and GPU model training.

### Offline and online augmentation

Similarly to the discussion of augmenting data on the CPU or on the GPU, another important consideration is to make sure the Data Augmentation is happening online, compared to offline. This refers to when the original instance is augmented in the data pipeline. Offline augmentation refers to augmenting the data and storing the augmented examples to the disk. Online augmentation describes augmenting the data as a new batch of the original data is loaded for a training step. We note that Online augmentation is much more powerful than Offline augmentation. Offline augmentation offers the slight benefit of faster loading times, but it does not really take advantage of the stochasticity and diversity enabled with most of the described augmentations.

Another important detail of this pipeline is augmentation multiplicity [128]. Augmentation multiplicity refers to the number of augmented samples derived from one original example. Fort et al. [128] and Hoffer et al. [129] illustrate how increasing augmentation multiplicity can improve performance. This approach could introduce significant memory overhead without an online augmentation pipeline. Additionally Wei et al. [130] point out that examples are often augmented online such that the model never actually trains with the original instances. Wei et al. propose separating the model into two fine-tuning heads, one which trains solely on the unaugmented data and the other trained on high magnitude augmentations. These works highlight the opportunity to explore fine-grained details in augmentation pipelines.

### Curriculum learning

Curriculum Learning describes having a human or meta-controller structured organization to the data batches. This includes varying the strength of Data Augmentation throughout training. Kucnik and Smith [131] find that it is much more efficient to subsample a portion of the dataset to be augmented, rather than augmenting the entire dataset. Wei et al. [132] demonstrate the efficacy of gradually introducing augmented examples to original examples in the training of triplet networks for text classification. We note this is very similar to our discussion of controllers for augmentation and searching for optimal magnitude and chaining parameters. Thakur et al. [85] describe that “selecting the sentence pairs is non-trivial and crucial for the success of the method”.

### Class imbalance

A prevalent issue explored in classification models is Class Imbalance [133]. In addition to customized loss functions, sampling techniques are a promising solution to overcome biases stemming from Class Imbalance. These solutions generally describe strategies such as random oversampling or undersampling [134, 135], in addition to interpolation strategies such as synthetic minority oversampling technique (SMOTE) [136]. SMOTE is a general framework to oversample minority instances by averaging between them. From the list of augmentations we have covered, we note that MixUp is very similar to this technique and has been explored for text data. It may be useful to use other techniques for oversampling to avoid potential pitfalls of duplicating instances.

## Discussion

### Task-specific augmentation for NLP

NLP encompasses many different task formulations. This ranges from text classification to paraphrase identification, question answering, and abstractive summarization, to name a few. The off-the-shelf Data Augmentation prescribed in the previous section will need slight adaptations for each of these tasks. For example, when augmenting the context in a question answering dataset, it is important to be mindful of removing the answer. The largest difference we have found between tasks from the perspective of Data Augmentation is that they vary massively with respect to input length. Short sequences will have to be more mindful of how augmentations change the original example. Longer sequences have more design decisions such as how to sample nested sentences for back-translation and so on. We refer interested readers to Feng et al. [16] who enumerate how Data Augmentation applies to summarization, question answering, sequence tagging, parsing, grammatical error correction, neural machine translation, data-to-text natural language generation (NLG), open-ended and conditional generation, dialogue, and multimodal tasks.

### Self-supervised learning and data augmentation

In both the case of self-supervised learning and Data Augmentation, we are looking to inject prior knowledge about a data domain. When a model is deployed, what is more likely: the data distribution changes or the task the model is supposed to perform with the data changes? In self-supervised learning, we look for ways to set up tasks and loss functions for representation learning. In Data Augmentation, we look for priors to manipulate the data distribution. A key advantage of Data Augmentation is that it is much easier to stack priors than self-supervised learning. In order to utilize multiple priors, self-supervised learning relies on highly unstable multi-task learning or costly multi-stage learning. In contrast, Data Augmentation only requires random sampling operations to integrate multiple priors.

We note that many of the key successes in self-supervised Learning rely on Data Augmentation, or have at least been dramatically improved by Data Augmentation. For example, the success of contrastive learning relies on Data Augmentation to form two views of the original instance. The most data-efficient GAN frameworks achieve data-efficiency through the use of Data Augmentation [137]. Further, DistAug [138] even tests Data Augmentation with large scale pixel autoregressive modeling in the ImageGPT model [139].

### Transfer and multi-task learning

Transfer learning has been one of the most successful approaches to training deep neural networks. This looks especially promising as more annotated datasets are collected and unified in dataset hubs. A notable example of which is HuggingFace datasets [140], containing 884 datasets at the time of this publication. In addition to transfer learning, researchers have additionally explored multi-task learning in which a model simultaneously optimizes multiple tasks. This has been well explored in T5 [141], which converts all tasks into language modeling. We believe there is room for Data Augmentation experiments in this space, such as the use of MixUp to combine data from multiple tasks or Back-Translation between curated datasets.

Wei et al. [130] propose an interesting extension, named as Multi-Task View (MTV), to the common practice of transfer learning to better utilize augmented subsets and share information across distributions. Multi-Task View (MTV) trains separate heads on augmented subsets and ensembles predictions for the final output. Geva et al. [142] have also shown utility in sharing a feature extractor base and training separate heads. In this case, Geva et al. train each head with a different task and reformulate inputs into unifying prompts for inference. Similar to the discussion of prompting under Generative Data Augmentation, there remains a significant opportunity to explore transfer learning, multi-task learning, and Data Augmentation.

### AI-GAs

One of the most interesting ideas in artificial intelligence research is AI-GAs (AI-generating algorithms) [10]. An AI-generating algorithm is composed of three pillars, meta-learning architectures, meta-learning the learning algorithms themselves, and generating effective learning environments. We believe that Data Augmentation and this interface to control data distributions will play a large role in the third pillar of generating learning environments. For example, embedding learning agents in teacher-student loops in which the teacher controls augmentation parameters to render the learning environment.

Learning the learning environment itself has been successfully applied to bipedal walking control with neural networks in POET [11]. POET is a co-evolutionary framework of control parameters and parameters that render walking terrains. Data Augmentation may be the most natural way of extending this framework to understanding language in which the environment searches for magnitude parameters of augmentation or subsets of data, as in curriculum learning. AI-GAs have been applied to vision problems in examples such as Generative Teaching Networks [12] and Synthetic Petri Dish [13]. In GTNs, a teacher network generates training data for a student network. Notably, the training data has high-frequency noise patterns that do not resemble natural image data. It could be interesting to see how well GTNs could generate text embeddings similar to the continuous optimization of prompt tuning.

## Conclusion

In conclusion, this survey has presented several strategies for applying Data Augmentation in Text data. These augmentations provide an interface to allow developers to inject priors about their task and data domain into the model. We have additionally presented how Data Augmentation can help simulate distribution shift and test generalization. As Data Augmentation for NLP is relatively immature compared to Computer Vision, we highlight some of the key similarities and differences. We have also presented many ideas surrounding Data Augmentation, from practical engineering considerations to broader discussions of the potential of data augmentation in building artificial intelligence. Data Augmentation is a very promising strategy and we hope our discussion section helps motivate further research interest.

## Data Availability

Not applicable.
